# Behaviors in Ethylene Polymerization of MgCl_2_-SiO_2_/TiCl_4_/THF Ziegler-Natta Catalysts with Differently Treated SiO_2_

**DOI:** 10.3390/molecules16021323

**Published:** 2011-01-28

**Authors:** Nichapat Senso, Bunjerd Jongsomjit, Piyasan Praserthdam

**Affiliations:** Center of Excellence on Catalysis and Catalytic Reaction Engineering, Department of Chemical Engineering, Faculty of Engineering, Chulalongkorn University, Bangkok 10330, Thailand

**Keywords:** Ziegler-Natta catalyst, polyethylene, silica, alkyl silane, XPS

## Abstract

The present research focuses on investigation of the catalytic behaviors of MgCl_2_-SiO_2_/TiCl_4_/THF Ziegler-Natta (ZN) catalysts with fumed SiO_2_ variously treated with silane compounds. The non-treated silica (NTS) and other silicas treated with dimethylsilicone fluid (TSDMSF), dimethyldichlorosilane (TSDMDCS), and hexamethyl-disilazane (TSHMDS) were employed. It was found that the Cat-TSDMDCS and Cat-TSHMDS exhibited remarkably high activity, even with a similar bulk Ti content as the others. Thus, the more powerful technique of XPS analysis was used to determine the Ti content at the catalyst surface. It was evident that the surface concentrations of Ti could play important role on the catalyst activity. As the result, the increased activity is proportional to the surface concentration of Ti. It was mentioned that the change in surface concentration of Ti with different treated silica can be attributed to the effect of silane spacer group and steric hindrance. The distribution of Ti on the external surface can be also proven by means of EDX mapping, which matched the results obtained by XPS analysis. The treated silica also resulted in narrower molecular weight distribution (MWD) due to the more uniform active sites produced. There was no significant change in polymer morphology upon treatment of the silica.

## 1. Introduction

In the polyolefin industry, the significant role of the Ziegler-Natta (ZN) catalysts is remarkable as are both industrial and academic interest in their reaction engineering [1,2,3,4]. In the production of polyolefins, the polymer particle morphology strongly affects the plant operation. The loss of polymer morphological control leads to many industrial operating problems, such as fouling and broadening reactor residence-time distribution. It is widely accepted that the polymer particle morphology is mainly determined by the morphology of a parent catalyst through the replication phenomenon [5,6]. The modification of supported-ZN catalyst is a preferred way to increase the morphology control of the polymers, increase the catalytic activity as well as increase catalytic stability.

Anhydrous magnesium dichloride (MgCl_2_) has been known as a preferred support for highly efficient ZN catalysts for the polymerization of olefins. MgCl_2_ is often convenient to use in producing ZN catalysts with good morphology and the high rates of polymerization activity [7,8]. However, the frailty of MgCl_2_ during preparation is still a problem for controlling the morphology of ZN when used in olefin polymerization. To overcome these problems, MgCl_2_-SiO_2_-bisupported titanium catalyst is preferred, especially when it is used in a gas-phase polymerization system [9,10,11]. For example, UCC has developed a series of MgCl_2_-SiO_2_/THF/TiCl_4_ four-component catalyst systems for gas-phase ethylene polymerization and ethylene/1-hexene copolymerization [12,13]. This catalyst exhibits good comonomer incorporation properties, and the polyethylene products show good morphology control. Somehow, the catalytic activity of MgCl_2_-SiO_2_/THF/TiCl_4_ system is lower than that without SiO_2_ added. To increase the catalytic activity, the properties of SiO_2_ such as type, shape, surface area [14] and OH content on the surface, should be considered in order to prepare a catalyst that is completely satisfactory for all purposes [15].

As is well known SiO_2_ has OH groups on the surface, which are a very important poison for ZN catalysts. Preheated SiO_2_ with different calcination temperatures (110–820 °C) is necessary to control the OH content on the surface of SiO_2_. The relationship between calcination temperature and TiCl_4_ reaction temperature was observed by Hornytzkyj *et al.* [16]. It was found that the lower reaction temperature of 175 and 125 °C led to amorphous titanium species, whereas the temperature of 350 °C or higher resulted in amorphous and agglomerated titanium species. The amount of amorphous titanium species present in the high temperature samples is a function of the reaction temperature of TiCl_4_ and of the preheat temperature of the SiO_2_ determined by etching with sulfuric acid. Consequently, suitable calcination methods can also decrease the OH groups on the surface, but it consumes much energy and increases the production cost, so chemical treatment is one of the promising ways to decrease the OH groups on the surface of SiO_2_ and it is practical. Hexamethyldisilazane (HMDS) and other organosilicon compounds (OSC), such as butyl dimethylsilyl (BDMS), dimethylsilicone fluid (DMSF), dimethyldichlorosilane (DMDCS), octadecyl dimethylsilyl (ODDMS) and trimethylsilyl (TMS) are commonly employed for the treatment of SiO_2_. This treated SiO_2_ has a wide variety of applications, including as a support for ZN catalysts. Although the properties of surface-modified solids can be predicted, observed changes often differ from expectations. Predictions of structural changes, for instance, are almost always related to primary particles [17,18,19], but structural changes of highly dispersed or porous solids are often hierarchal. In the case of fumed oxides, structures are dictated by aggregates of primary particles and agglomeration of aggregates. This hierarchal structure is difficult to predict, as it is dependent upon a balance of forces, which are altered as a result of surface modification [20,21]. This often results in unexpected changes to the modified surface of SiO_2_. Hertl and Hair [22] studied the treatment of silica with HMDS and used it as a support in ZN catalyst. They proposed that the reaction of HMDS occurs almost exclusively with isolated OH groups, leaving the H-bonded OH groups unoccupied. This property of HMDS has been applied successfully to study the bi-functional reactivity of TiCl_4_ toward H-bonded OH groups on silica in both vapor phase [22] and organic solution [23]. There were no changes of HMDS coverages on the HMDS-modified silica after the reaction with TiCl_4_ at 175 °C [24]. Instead, the number of titanium atoms were half of that reacted without silylation, and the reaction led exclusively to doubly bonded titanium species. Consequently, the attainable trimethylsilyl surface coverage was not only determined by the steric hindrance, but also by the lower reactivity of HMDS toward H-bonded in OH groups. However, from the previous research, there is still little information about the effects of SiO_2_ treated with different functional ethyl groups, such as dimethylsilicone fluid (DMSF), dimethyldichlorosilane (DMDCS) and hexamethyldisilazane (HMDS) on ZN catalyst properties and polymer properties although this information is of interest in both academia and industryl, especially in the surface study area. The XPS, SEM and EDX techniques were used in this investigation. Four types of CAB-O-SIL fumed silica were chosen for study based on the fact that they have well defined surface structures. They can be prepared by reproducible procedures and they are commonly used as catalyst supports. In this study, the influence of different silane compounds employed for the treatment of fumed silica in MgCl_2_-SiO_2_/TiCl_4_/THF catalysts on ethylene polymerization was examined The properties obtained were also determined and are discussed in detail.

## 2. Results and Discussion

**Figure 1:**
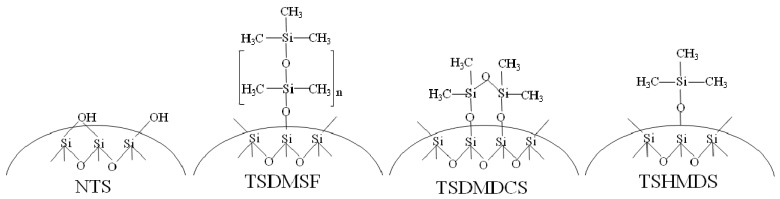
Different organo-silicon groups on the surface of variously treated fumed silicas.

The structures of various treated fumed silicas used as supports in ZN catalysts are shown in Figure 1. There were four modified surface fumed silicas: (i) untreated SiO_2_ (NTS); (ii) SiO_2_ treated with dimethylsilicone fluid (TSDMSF); (iii) SiO_2_ treated with dimethyldichlorosilane (TSDMDCS); and (iv) SiO_2_ treated with hexamethyldisilazane (TSHMDS).

**Figure 2:**
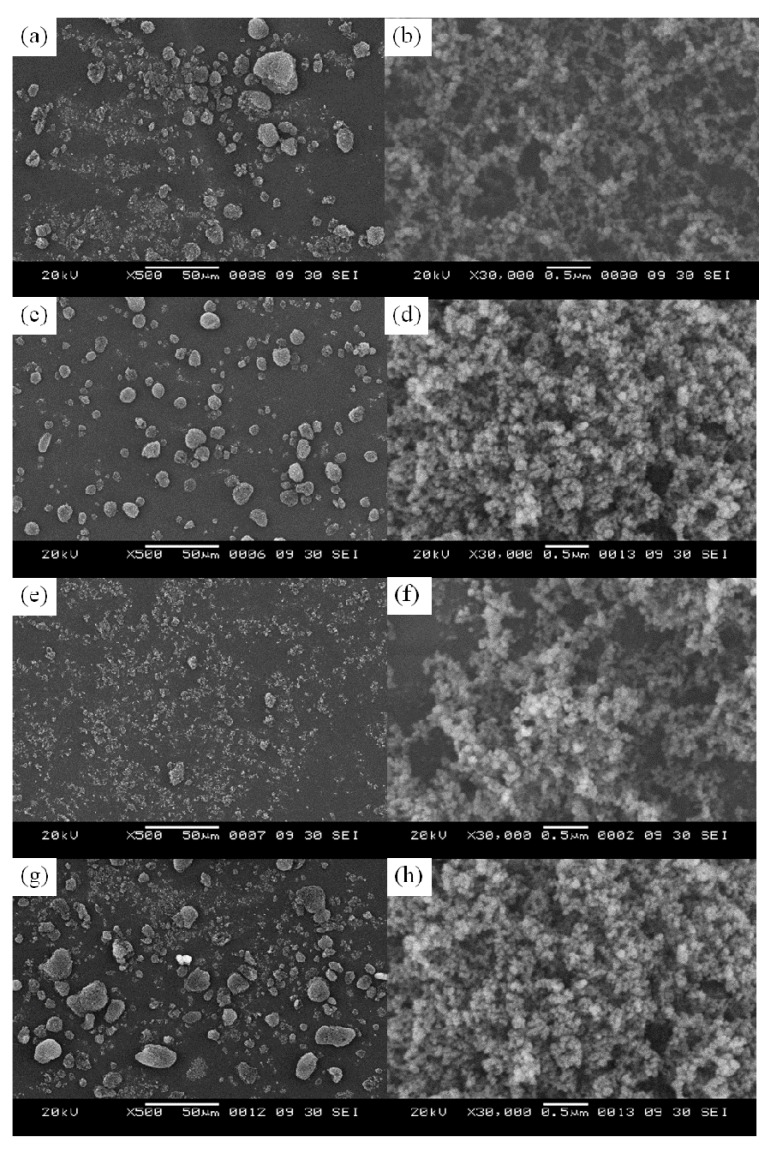
SEM images of different treated silicas; **(a)** NTS; **(c)** TSDMSF; **(e)** TSDMDCS; **(g)** TSHMDS; and **(b)**, **(d)**, **(f)**, and **(h)** represent the primary particles of corresponding treated silicas, respectively.

The SEM images of all the samples are shown in Figure 2. It can be seen that the primary particles of fumed SiO_2_ [Figure 2(b), (d), (f), and (h)] display no change in shape and size after treatment. A little agglomeration of particles occurred in TSDMSF and TSHMDS. The agglomeration of TSDMSF and TSHMDS particles results from the balance of force and type of chemical treatment. This result is supported by other research [20,21].

After making the ZN catalysts with different treated SiO_2_ supports, the bulk Ti content for all catalysts (Cat-NTS, Cat- TSDMSF, Cat-TSDMDCS, and Cat-TSHMDS) was analyzed by ICP. The results are shown in Table 1.

It was found that the Ti contents in all catalyst samples were similar and within the 2.11–2.34 wt % range. Then, all catalyst samples were tested for ethylene polymerization under the specified conditions. The activity results are also shown in Table 1. It can be observed that the silica treated with silane compounds tends to show increased catalytic activity due to the effect of the spacer groups introduced by silane treatment [25]. It was also surprising that although all catalysts had similar amount of Ti contents in bulk, they exhibited different catalytic activity. As seen, Cat-TSDMDCS, and Cat-TSHMDS exhibited the remarkably high activity (about four times higher than Cat-NTS). For elucidation, another parameter such as the Ti content at surface needed to be verified. One of the most powerful techniques used to determine the surface properties is X-ray photoelectron spectroscopy (XPS). The oxidation state related to the binding energy of Ti and other elements was evaluated. Ti exhibited its binding energy at ca. 459 eV, indicating the Ti 2p state in all catalyst samples.

The surface concentrations obtained from the XPS measurements for Ti and other elements are also shown in Table 2. It was found that the surface concentrations for Ti 2p in the Cat-TSDMDCS and Cat-TSHMDS samples were remarkably high. On the other hand, for both samples Ti was located on the outer surface of the catalysts. This is probably due to less steric hindrance of the Cat-TSDMDCS and Cat-TSHMDS samples compared to the Cat-TSDMSF sample, as seen in Figure 1. The large amounts of Ti located on the surface are the main reason for the very high activities obtained from the Cat-TSDMDCS and Cat-TSHMDS samples as seen in Table 1. Therefore, the high activity of the ZN catalysts can be attributed to the large amounts of Ti content on surface, not in the bulk of catalysts.

**Figure 3:**
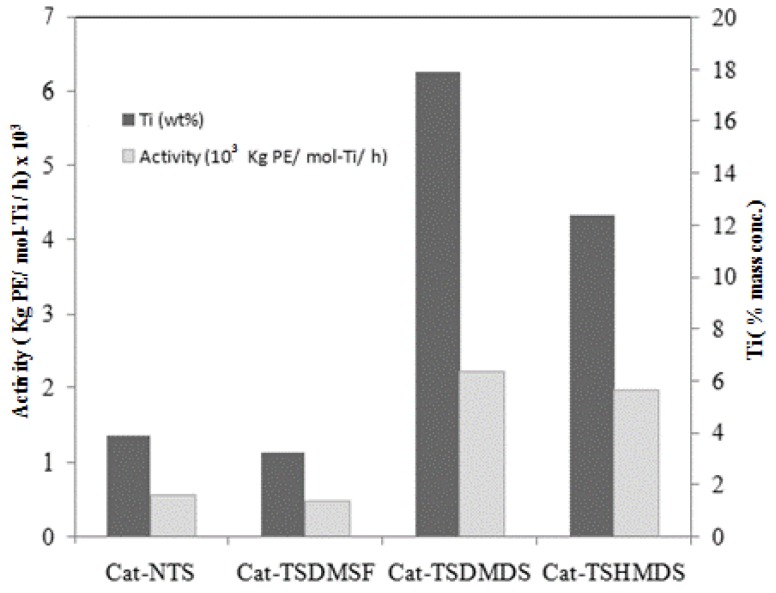
Relationship between the surface concentrations of Ti and the activities of catalysts.

In order to illustrate the relationship between the surface concentrations of Ti and the activities of catalysts, Figure 3 is constructed. It can be seen that the activities of catalysts are proportional to the surface concentrations of Ti present on the catalyst, as mentioned before.

Besides XPS measurements, the other powerful techniques used to determine the morphology and elemental distribution are SEM and EDX, respectively.

**Figure 4:**
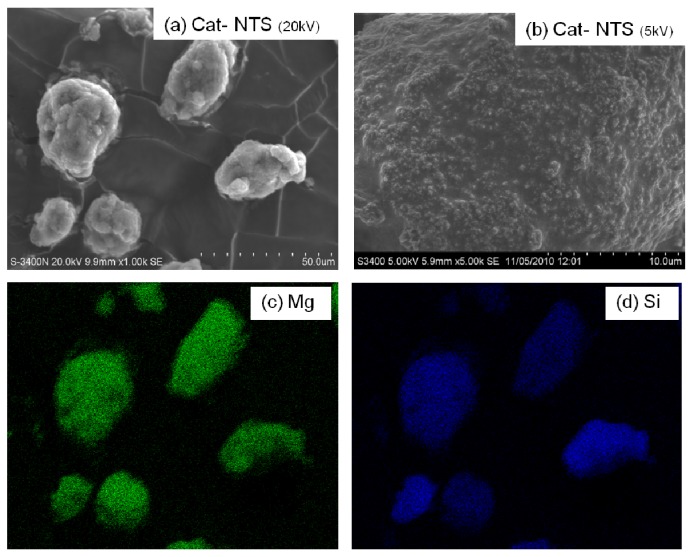

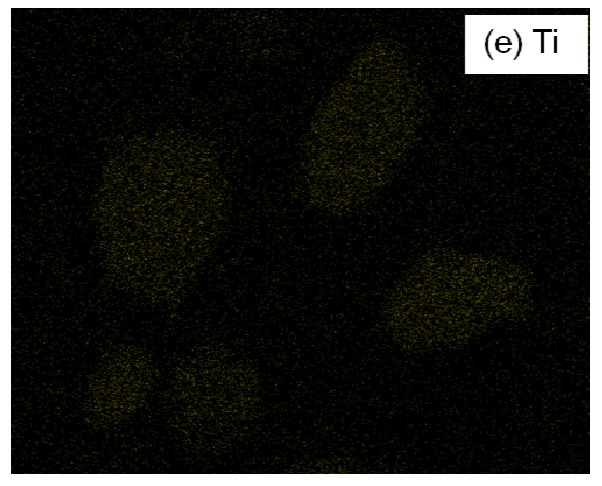
SEM micrograph and elemental distribution on Cat-NTS; **(a)** SEM image; **(b)** external surface; **(c)** Mg distribution; **(d)** Si distribution and **(e)** Ti distribution.

**Figure 5:**
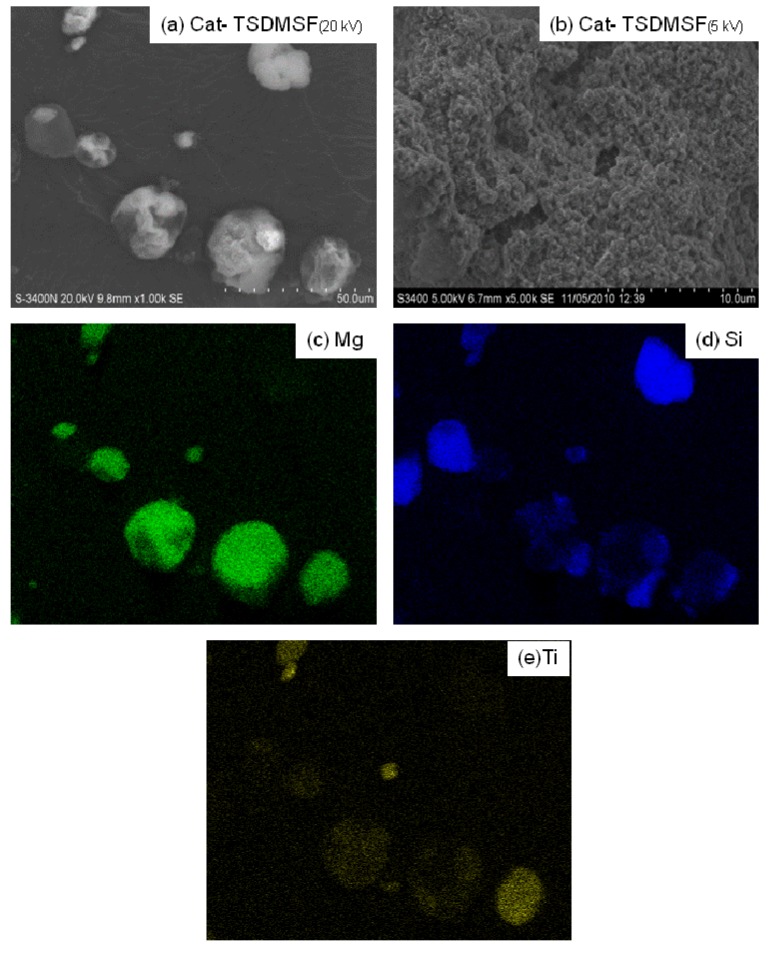
SEM micrograph and elemental distribution on Cat-TSDMSF; **(a)** SEM image; **(b)** external surface; **(c)** Mg distribution; **(d)** Si distribution and **(e)** Ti distribution.

**Figure 6:**
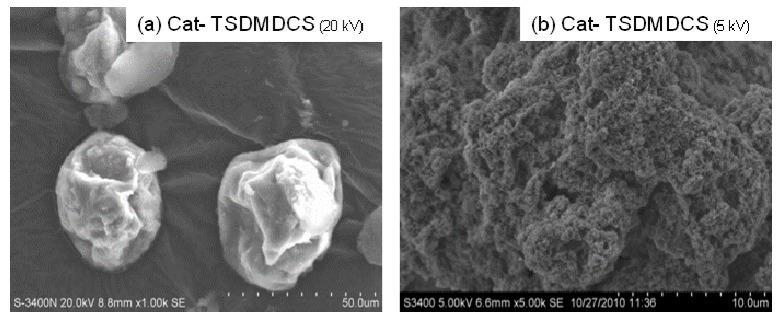

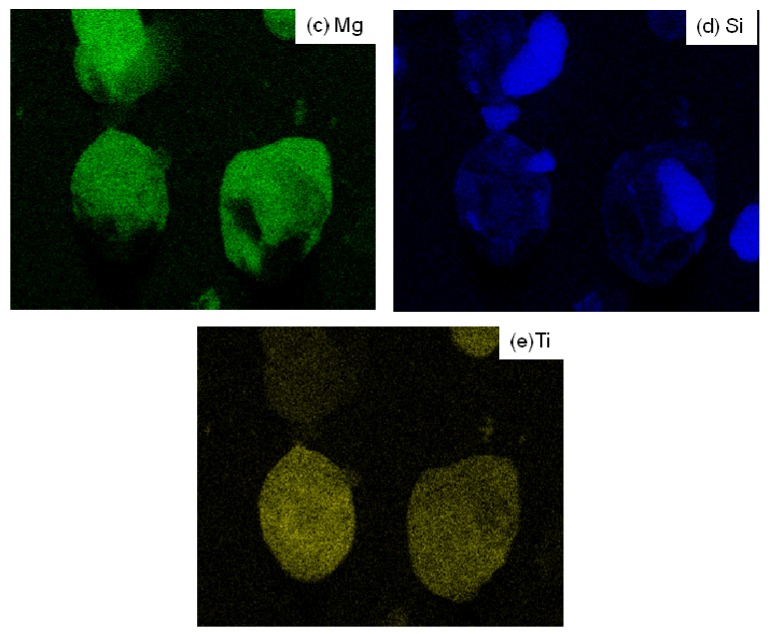
SEM micrograph and elemental distribution on Cat-TSDMDCS; **(a)** SEM image; **(b)** external surface; **(c)** Mg distribution; **(d)** Si distribution and **(e)** Ti distribution

The SEM and EDX mapping for all catalyst samples are shown in Figure 4 to Figure 7 displaying the external surface of the catalysts and distribution of Mg, Si, and Ti on them. It should be mentioned that EDX only measures the concentrations in a layer less than 1 micrometer from the surface [26,27]. As a matter of fact, for whole catalyst particles, EDX measures the concentration on external surface of the particles. Figure 4, Figure 5, Figure 6 and Figure 7 were all obtained with identical magnification. Considering the Ti distribution on the external surface of each catalyst represented by yellow patches (e), it can be clearly seen that the intensities of yellow patches (Ti at surface) in Figure 4 (Cat-NTS) and Figure 5 (Cat-TSMDSF) are very low compared with those in Figure 6 (Cat-TSDMDCS) and Figure 7 (Cat-TSDMDCS) corresponding to the XPS measurements as mentioned before. Therefore, both XPS and EDX mapping results can be used to confirm the rich Ti surface contents on the Cat-TSDMDCS and Cat-TSDMDCS samples leading to high catalytic activity. It is known that generally, TiCl_4_ can bind to OH groups in the untreated silica. However, after treatment, it is mostly located on the MgCl_2_ support as seen in the EDX mapping.

The molecular weight (M_w_) and molecular weight distribution (MWD) of polymers obtained from different catalysts as analyzed by GPC are listed in Table 3. It can be seen that Cat-NTS produced polymer having the highest M_w_, M_z_, and MWD compared to polymers obtained from other catalysts. The broad MWD for Cat-NTS can be also attributed to the high molecular weight tail (M_z_). It can be observed that all treated silica for ZN catalysts apparently resulted in narrower MWD, as also reported in our previous work [25]. It was suggested that the treatment of silica with silane can result in more uniform active centers leading to narrower MWD.

A typical SEM micrograph of polymer obtained from all catalysts, which was similar, is shown in Figure 8. Hence, it indicates that the treated silica has no effect on the polymer morphologies.

## 3. Experimental

### 3.1. Chemicals

Polymerization grade ethylene and triethylaluminum (TEA) donated by PTT Chemical Plc., were used without further purification. TiCl_4_ (Aldrich) and MgCl_2_ (anhydrous) were donated by Toho Catalyst Co., Ltd. Fumed silica [non-treated (NTS), surface area 149.35 m^2^/g] and silica treated with different organic compounds [dimethylsilicone fluid (TSDMSF, surface area 121.60 m^2^/g), with dimethyldichlorosilane (TSDMDCS, surface area 112.00 m^2^/g), and with hexamethyldisilazane (TSHMDS, surface area 130.95 m^2^/g)] were supplied by Cabot Corporation and average particle size of all silicas were 0.2–0.3 µm as reported by the supplier. All types of silica were heated under vacuum at 120 °C for 2 h. Hexane and tetrahydrofuran were dried over dehydrated CaCl_2_ and distilled over sodium benzophenone under an argon atmosphere prior to use. Ultra high purify (UHP) argon (99.999%) was purchased from Thai Industrial Gas Co., Ltd. and was further purified by passing through 3 Å molecular sieves., BASF catalyst R3-11G, NaOH and phosphorus pentaoxide (P_2_O_5_) to remove traces of oxygen and moisture. All chemicals were manipulated under an inert atmosphere using a vacuum glove box and Schlenk techniques.

### 3.2. Catalyst Preparation

The catalyst was prepared in a 500 mL vessel equipped with temperature control, and a turbine agitator. First, anhydrous tetrahydrofuran (150 mL) was added into the vessel and heated up to a 50 °C. Then, magnesium metal (0.12 g) was added, followed by titanium tetrachloride (2 mL). The mixture was continuously agitated and the temperature was held at about 70 °C. After that, magnesium dichloride (4.5 g) was added, and the heating process was continued at 70 °C for another 3 h. Then different treated silicas (NTS, TSDMSF, TSDMDCS and TSHMDS, 4.5 g) were slowly added to the mixture, which was stirred for 1 h to thoroughly disperse the silica in the solution. The temperature of mixture was held at 70 °C throughout this period and an argon atmosphere was maintained for all time. This mixture was washed, and then dried under vacuum.

### 3.3. Polymerization Reaction

Ethylene polymerization was carried out in a 100 mL stainless steel autoclave reactor equipped with magnetic stirrer. The prescribed amount of hexane (30 mL), TEA and the SiO_2_-MgCl_2_-supported ZN catalysts, such as Cat-NTS, Cat-TSDMSF, Cat-TSDMDCS and Cat-HMDS (Al/Ti molar ratio = 100) were added into the reactor. The ethylene pressure and reactor temperature were kept constant during polymerization [pressure in reactor = 50 psi and polymerization temperature was held at 80 °C]. Due to the fixed ethylene consumption (at 0.018 moles), the polymerization time was defined as the time that all ethylene gas was totally consumed [the equivalent pressure drop of 42 kPa (6 psi) was observed]. The polymerization time was recorded to calculate the activity. The reaction was terminated by adding acidic methanol and polymer was stirred for 30 min. After filtration, the polymer obtained was washed with methanol and dried at room temperature.

### 3.4. Polymer and Catalyst Characterization

#### 3.4.1. Scanning electron microscopy and energy dispersive X-ray spectroscopy (SEM/EDX)

The morphological observations of polymers were carried out with a JEOL JSM-6400 scanning electron microscope. Micrographs were taken at 5-kV and 20-kV acceleration voltage. Before scanning electron microscopy (SEM) observations, the fracture surfaces of blends were coated with a thin layer of gold to avoid electrical charging and increase contrast during observation. The EDX was performed using Link Isis series 300 program, to determine the elemental distribution in catalysts.

#### 3.4.2. Inductively coupled plasma (ICP)

Titanium content was measured using inductively coupled plasma atomic emission spectroscopy equipment (ICP-OES optima 2100 DV from PerkinElmer). In order to digest the sample, the catalyst was dissolved in hydrofluoric acid. The mixtures were stirred over a night. After the catalyst was completely dissolved, the solution was diluted with ID water to a volume of 100 mL.

#### 3.4.3. X-ray photoelectron spectroscopy (XPS)

The chemical states and surface concentration of the elements were measured by the XPS technique using an Amicus photoelectron spectrometer with Mg K_α_ X-ray source at current of 20 mA and 10 keV, resolution of 0.1 ev/step, and pass energy of 75 eV. The binding energy was calibrated by the C 1s peak at 285.0 eV. In this study, the sample was always treated under argon to prevent the sample from damage by moisture and oxygen in the atmosphere.

#### 3.4.4. Gel permeation chromatography (GPC)

A high temperature GPC (Waters 150-C) equipped with a viscometric detector, differential optical refractometer and four Styragel HT type columns (HT3, HT4, HT5, and HT6) with 1 × 107 exclusion limit for polystyrene was used to determine the molecular weight (M_W_) and molecular weight distributions (MWD) of the polymers produced. The analyses were performed at 140 °C using 1, 2, 4-trichlorobenzene as the solvent. The columns were calibrated with standard narrow MWD polystyrene.

## 4. Conclusions 

In summary, the catalytic behaviors of MgCl_2_-SiO_2_/TiCl_4_/THF catalysts with different treated silicas, such as Cat-NTS, Cat-TSDMSF, Cat-TSDMDCS, and Cat-TSHMDS were investigated. Although all catalysts exhibited the similar bulk Ti content, their activities were different. Therefore, the measurement of surface concentrations of Ti by means of XPS techniques was crucial. It was found that Cat-TSDMDCS and Cat-TSHMDS rendered high activities due to the large amounts of Ti at the surface of the catalyst. This could be also confirmed by the EDX mapping of Ti on the external surface. It can be concluded that for each catalyst the increased activity is proportional to the surface concentration of Ti. It is worth noting that the increased activity for the treated silica for Cat-TSDMDCS and Cat-TSHMDS catalysts can be attributed to both the effects of the silane spacer group and less steric hindrance. The treated silica in MgCl_2_-SiO_2_/TiCl_4_/THF catalysts apparently resulted in narrower MWD due to the increased uniformity of the active sites. However, there was no significant change in polymer morphology with the treated silicas as seen by SEM.

## Figures and Tables

**Figure 7:**
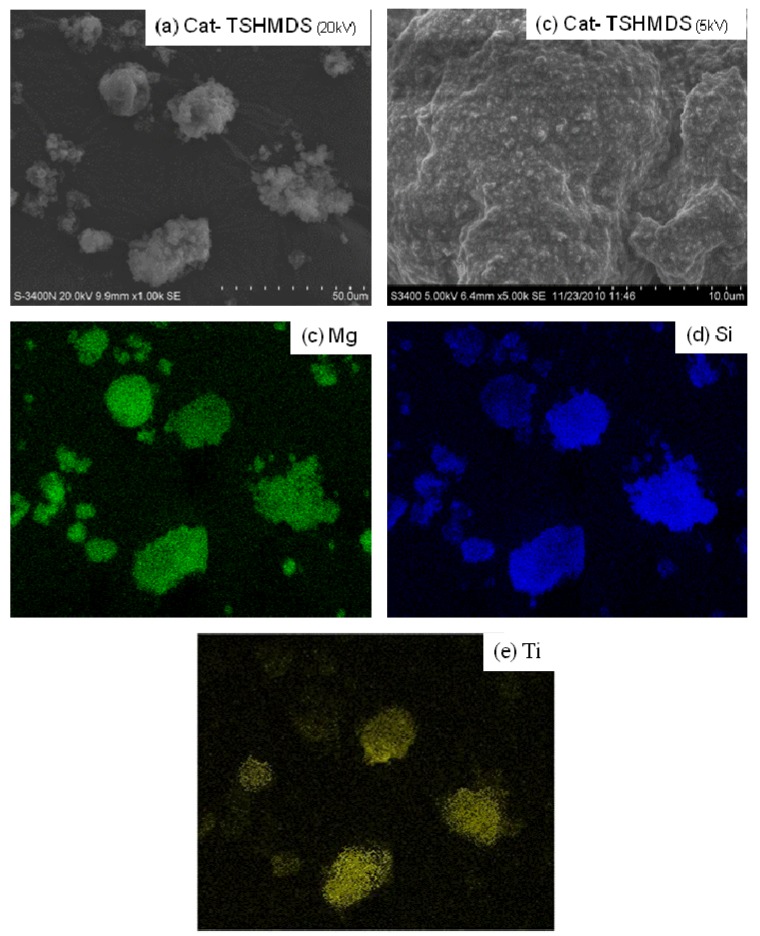
SEM micrograph and elemental distribution on Cat-TSHMDS; **(a)** SEM image; **(b)** external surface; **(c)** Mg distribution; **(d)** Si distribution and **(e)** Ti distribution.

**Figure 7:**
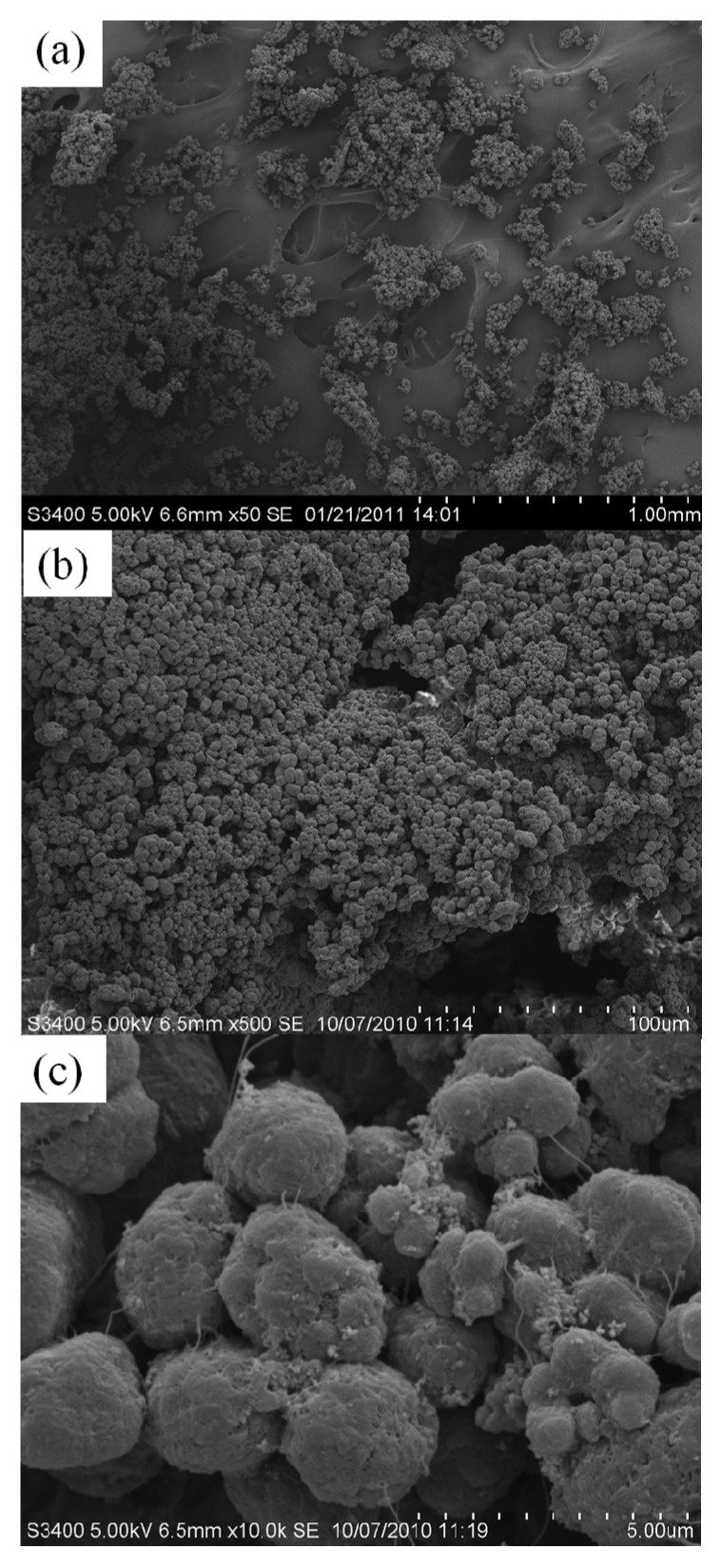
A typical SEM micrograph of polyethylene samples of the treated silica ZN catalyst; **(a)** polyethylene at × 50 magnification; **(b)** polyethylene at × 500 magnification; and **(b)** surface of polyethylene at × 10k magnification.

**Table 1:** Ti content in bulk and activity of different catalysts.

Sample	Treatment	Ti in bulk of catalysts (wt %) ^a^	Activity ^b^ (kg PE/mol-Ti/h)
Cat-NTS	Untreated silica	2.27	1,570
Cat-TSDMSF	Dimethylsilicone Fluid	2.32	1,370
Cat-TSDMDCS	Dimethyldichlorosilane	2.34	6,370
Cat-TSHMDS	Hexamethyldisilazane	2.11	5,620

^a^ Obtained by ICP analysis; ^b^ Ethylene polymerization at 50 psi, 80 °C, Al/Ti = 100.

**Table 2:** Surface concentrations of Ti, Si and Mg in all catalysts obtained by XPS analysis.

Peak	Cat-NTS	Cat-TSDMSF	Cat-TSDMDCS	Cat-TSHMDS
Ti2p	3.10	3.22	17.90	12.40
Si2p	64.05	88.55	37.81	56.13
Mg2s	32.85	8.23	44.30	31.47

**Table 3:** Molecular weights and their distribution of polymers obtained from different catalysts.

Sample	M_n_ (kg/mol)	M_w_ (kg/mol)	M_z_ (kg/mol)	M_v_ (kg/mol)	MWD (M_w_/M_n_)
Cat-NTS	40	1,028	4,574	739	25.7
Cat-TSDMSF	23	359	4,073	339	15.6
Cat-TSDMDCS	55	787	3,086	595	14.3
Cat-TSHMDS	24	437	4,064	405	18.2
